# Chemical Characterization, Antidiabetic and Antioxidant Potential of *Allium cepa* Peels Silver Nanoparticles Against Alloxan Induced Diabetes in Rabbits

**DOI:** 10.1002/fsn3.70518

**Published:** 2025-06-27

**Authors:** Sadia Batool, Mazhar Abbas, Sidra Ashraf, Muhammad Kamran Rafique, Aqsa Mumtaz, Tariq Hussain, Muhammad Riaz, Ghulam Rasool, Muhammad Adnan Ayub, Quzi Sharmin Akter

**Affiliations:** ^1^ Department of Basic Sciences (Section Biochemistry) University of Veterinary and Animal Sciences Lahore (Jhang Campus) Jhang Pakistan; ^2^ Department of Pathobiology (Section Parasitology) University of Veterinary and Animal Sciences Lahore (Jhang Campus) Jhang Pakistan; ^3^ Department of Basic Sciences (Section Pharmacology and Toxicology) University of Veterinary and Animal Sciences Lahore (Jhang Campus) Jhang Pakistan; ^4^ Department of Allied Health Sciences University of Sargodha Sargodha Pakistan; ^5^ Department of Chemistry University of Sahiwal Sahiwal Pakistan; ^6^ Department of Genetics and Animal Breeding, Faculty of Animal Science and Veterinary Medicine Patuakhali Science and Technology University Patuakhali Bangladesh

**Keywords:** AgNPs synthesis, *Allium cepa* peels, antioxidant levels, blood glucose levels, diabetes, LCMS‐MS, XRD

## Abstract

Diabetes is one of the major causes of death worldwide. Onion peels have antioxidant and antidiabetic activities due to the presence of flavonoids and phenolic compounds. In current research, the antidiabetic potential of a herbal‐based silver nanodrug synthesized using aqueous extract of *Allium cepa* peels was studied against Alloxan‐induced diabetes in rabbits. The green synthesized reduced silver nanoparticles (AgNPs) and aqueous extract were characterized using X‐ray diffraction analysis (XRD) and liquid chromatography mass spectrometry (LCMS‐MS) analysis. After the acclimation period, the rabbits were divided into nine groups of 5 rabbits each (*n* = 5). Experimental animals were injected with a single dose of Alloxan monohydrate (160 mg/mL per kg BW) and then orally treated with aqueous extract of *A. cepa* peels and AgNPs at three dose concentrations (100, 200, 300 mg/mL per kg BW) for 20 days. Serum levels of blood sugar, renal function tests, lipid profile, and oxidative stress markers were determined photometrically. Results from mass spectral analysis revealed that quercetin and vanillic acid are the major phenolic compounds. XRD analysis confirmed the stabilization and shape of green synthesized AgNPs. Results from in vivo studies indicated that treatment with aqueous extract of *A. cepa* peels and AgNPs attenuated the Alloxan‐induced diabetes in a dose‐dependent manner, as demonstrated by a decrease in blood sugar, triglycerides, urea, creatinine, and cholesterol to 100 ± 0.8, 57.66 ± 0.8, 38 ± 0.8, 1.11 ± 0.08, 40 ± 0.00 mg/dL, respectively, in rabbits administered with 300 mg/mL per kg BW AgNPs. The levels of oxidative stress markers (CAT, SOD, and GPx) in blood, that is, 3.6 ± 0.08, 6.0 ± 0.08, 918 ± 2.0 IU/mL, respectively, were also increased in rabbits administered with 300 mg/mL per kg BW AgNPs. Our results conclude that synthesized AgNPs using aqueous extract of *A. cepa* peels have a protective effect against Alloxan‐induced diabetes.

## Graphical abstract

The study demonstrated remarkable antidiabetic potential of *A. cepa* peels extract and the synthesized silver nanoparticles as evidenced through the lowered glucose, cholesterol, and triglyceride levels while increasing antioxidant enzyme activities in *A. cepa* extract and synthesized nanoparticle‐treated animals. *A. cepa* peels as a promising competitor for the treatment of type 2 diabetes mellitus with high therapeutic efficiency and lower side effects.

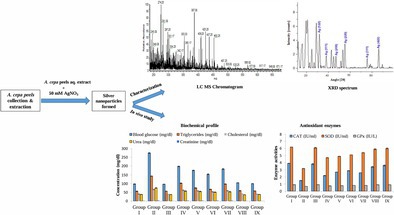

## 1 Introduction

Diabetes mellitus (DM) is a metabolic disorder associated with impaired signaling & resistance of insulin, and β‐cell dysfunction, increased oxidative stress, sub‐clinical inflammation, abnormal glucose and lipid metabolism (Airaodion et al. 2019; Summer et al. 2024). The causative agents include dithizone, monosodium glutamate, gold thioglucose, high fructose load, high glucose load and anti‐insulin serum. Alloxan monohydrate and streptozotocin (STZ) are frequently used in diabetes research (Macdonald Ighodaro et al. 2017). Different factors contribute to the development of diabetes, but oxidative stress is one among the major factors. Alloxan monohydrate along with its byproducts like dialuric acid are selectively accumulated in beta cells as glucose analogue and mediates oxidative stress by producing reactive oxygen species (ROS) causing the apoptotic death of β‐cell of pancreas which in turn leads to the reduction in insulin production. Further, Alloxan monohydrate increases the concentration of calcium ions (Ca^2+^) in beta cells of pancreatic islets which contributes to the abnormal release of insulin and ultimately cause the death of beta cells of pancreatic islets (Rohilla and Ali 2012).

Diabetic patients are given oral hypoglycemic or insulin for the control of blood sugar, but these have side effects as well. Natural fresh fruits and vegetables reduce the risk of chronic disorders due to the presence of variety of bioactive phytochemicals. Besides this, processing of agricultural products produce large quantities of byproducts which are usually discarded as waste while these products are the rich source of bioactive ingredients (Kumar et al. 2022; Riaz et al. 2023). Onion peel's yield is more than 500,000 tones which is being dumped as waste, whereas these are rich in phytoconstituents like phenolics (vanillic acid and Ferulic acid), flavonoids (Quercetin), flavanols, anthocyanins, and tannins (Stoica et al. 2023), as compared to onion bulb. Owing to these bioactive composition, *Allium cepa* peels can be used as health promoting ingredient especially in biomedical and pharmacological fields due to their anti‐oxidant, anti‐cancer, anti‐microbial, anti‐obesity, neuroprotective, cardioprotective, and anti‐diabetic properties (Kumar et al. 2014).

The main disadvantage of medications derived from herbs was their low bioavailability. Nanotechnology is an interdisciplinary field that can manipulate the matter at nanoscale and the nanoparticles can revolutionize various fields through its application in medical sciences, material sciences, energy and electronics (Gupta et al. 2024; Idris and Roy 2024a, 2024b). Nanoparticles consist of inorganic, organic and metallic substances, and can be prepared through different methods, such as hydrothermal synthesis, thermal decomposition and chemical vapor deposition (Haque et al. 2024). Metallic nanoparticles are advantageous due to their reactivity, large surface area and catalytic power which make them highly important for multiple applications. Silver nanoparticles have wide spread uses across industries like pharmaceuticals, cosmetics, ceramics, paints, textiles, energy and agriculture due to their biocompatibility, stability, eco‐friendliness, non‐toxicity, hydrophilicity, heat resistance and antimicrobial properties (Idris, Roy, Malik, et al. 2024; Naik et al. 2023; Vu et al. 2022). According to Zhang et al., nanoparticles hold great potential in addressing the inadequate bioavailability of herbal‐based medications (Zhang et al. 2019). Natural extract of plants acts as chelating agents for the favorable synthesis of metal nanoparticles. Silver nanoparticles (AgNPs) are the most beneficial of all the metallic nanoparticles because of their ability to transport therapeutic molecules that bound to surfaces and provide guidance about the fundamental functions and biological behaviors of disease cells. These green synthesized nanosized AgNPs are marked as antioxidants, genoprotective, antimicrobial, antianalgesic, antitumor, anti‐inflammatory, anti‐infections and anticancer agents (Bhusari et al. 2023; Idris, Roy, Subramanian, et al. 2024; Tahir et al. 2024). To address safety concerns, nanoparticles must be characterized before being used in nanomedicines (Zhang et al. 2016). To the best of our knowledge, *A. cepa* peels extract and its synthesized nanoparticles have been chemically characterized. However, no thorough phyto‐chemical and antidiabetic data about silver nanoparticles synthesized using aqueous extract of *A. cepa* peels in particular against Alloxan monohydrate induced diabetes is available so far. In the present study, we aim to characterize the phytochemical profile of AgNPs produced using aqueous extract of *A. cepa* peels. Furthermore, investigating the in vivo protective capability of AgNPs against Alloxan induced chronic diabetes in rabbits through the determination of biochemical markers.

## 2 Materials and Methods

### 2.1 Chemicals and Reagents

Silver nitrate and all other chemicals used in this research were of analytical grade and purchased from Merck and Sigma Aldrich. The Randox kits to test blood sugar levels, renal function tests, lipid profile, and oxidative stress markers were purchased from MyBiosource, USA.

### 2.2 Collection and Preparation of Aqueous Extract

*Allium cepa* peels were obtained from the local market. The obtained peels were washed using distilled water, and then shade dried. Later, these dried peels were ground to a fine powder and soaked in aqueous medium. The mixture was agitated and filtered to obtain aqueous extract. Finally, the filtrate was concentrated on boiling water bath for 24 h. The resulting colloidal solution was lyophilized to get dried powder of aqueous extract of *Allium cepa* peels and stored at 4°C for further analysis (Abdalla et al. 2022).

### 2.3 Green Synthesis of Silver Nanoparticles (AgNPs)

**FIGURE 1:**
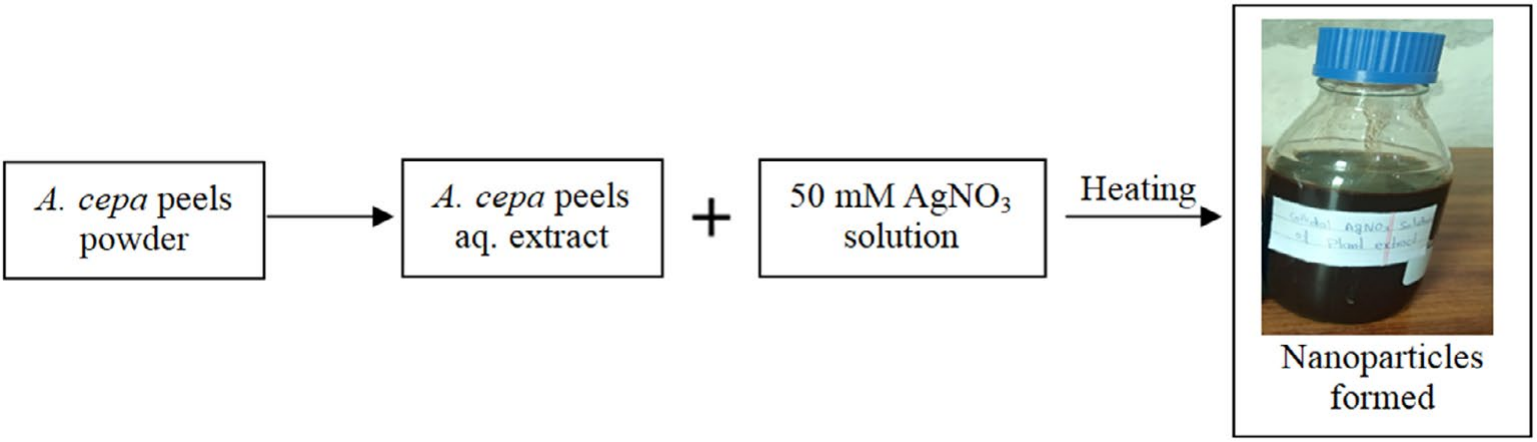
Flow chart for the formation of silver nanoparticles.

Green synthesis was carried out by mixing the 100 mL of 50 mM AgNO_3_ solution with 400 mL of prepared *A. cepa* peels aqueous extract under continuous stirring in a 1000 mL Erlenmeyer conical flask. After 2 h, synthesis of AgNPs was visually observed by color change (Figure 1). Finally, the obtained color reaction mixture was lyophilized and a violet‐brown hygroscopic powder was stored in dark for further characterization (Saratale et al. 2020).

### 2.4 Characterization

#### 2.4.1 LCMS Analysis of Aqueous Extract of *A. cepa* Peels

Phytoconstituents present in aqueous extract of *A. cepa* peels was assessed and quantified by Liquid Chromatography coupled with Mass Spectrometry. Linear Ion Trap Mass spectrometer model; LTQ XL (Thermo Electron Scientific, USA) was equipped with electro spray ionization (ESI) source with Capillary voltage of 4.7 kV. Methanol was used as mobile phase in columns. Sample was prepared by following solid phase extraction (SPE) method, using acetonitrile as solvent and filtered by syringe filter paper with pore size of 0.2 μm. A 10 μL of prepared sample was injected by direct insertion method with the help of syringe columns and instrument was set at Capillary temperature of 295°C, Sheath gas flow rate of 17 units, and Auxiliary gas flow rate of 7 units. Various peaks were selected for fragmentation MS/MS using Xcalibur 2.0.7 Software, with collision induced dissociation (CID) energy ranging from 20 to 30. Compounds were identified in both negative and positive scan ion modes in scanning mass range of 50–2000 m/z (El‐Askary et al. 2019).

#### 2.4.2 XRD Analysis of Green Synthesized AgNPs

To carry out XRD analysis, Braker Diffractometer (Model‐D2 Phaser XE‐T Edition) was used. Crystallinity of green synthesized AgNPs was analyzed by using Cu‐Kα (*λ* = 1.5406) as the target radiation source. Diffractograms were recorded at 2*ϴ* range between 30° and 80° with an intensity of 0–150 counts (Narayanasamy et al. 2020). Average crystalline size of synthesized AgNPs was evaluated by using Scherer's equation:

D=Kλβ12cosθ

Here *D* is the average crystalline size, *K* is the constant, *λ* is the wavelength, *β* is full width at half maximum, and *θ* is Bragg angle (Rashid et al. 2019).

### 2.5 In Vivo Experimental Investigation

The experiment was carried out on 45 male rabbits with average BW of 1 kg after approval from Ethical Committee. The experimental animals were purchased from local market and housed in steel cages under standard conditions of temperature, light, and ventilation, for 2 weeks before experimental work. Animals were accessed to fed on normal standard diet. After that, they were allowed to fasten overnight, diabetes mellitus was induced in experimental rabbits by IP injection of a single dose of Alloxan monohydrate (160 mg/mL per kg BW), except negative control rabbits which were fed with normal healthy diet throughout the experimental duration. After 72 h of diabetes induction, biochemical assessment was done and recorded to confirm the diabetes in experimental animals. Then animals were divided into nine groups (*n* = 5) and differentiated by ear tagging. The experimental protocol was followed as described earlier by (Zhang et al. 2019). The treatment of animals of all the groups was carried out as: The Group‐I rabbits were treated orally with Normal healthy diet while Group‐II rabbits were marked as Positive control group and were not given any treatment after the induction and confirmation of diabetes. Although Group III rabbits were marked as drug control rabbits and were treated with Giblencelamide in the oral dose of 5 mg/20 mL per kg BW. Group IV, V and VI were low (100 mg/mL per kg BW/day), medium (200 mg/mL per kg BW/day) and high dose (300 mg/mL per kg BW/day) aqueous extract of *Allium cepa* peels orally treated rabbits, respectively. Similarly Group VII, VIII and IX were low (100 mg/mL per kg BW/day), medium (200 mg/mL per kg BW/day) and high dose (300 mg/mL per kg BW/day) Green synthesized AgNPs orally treated rabbits, respectively. The treatment of animals of Group III, IV, V, VI, VII, VIII and IX was lasted for 20 days after the confirmation of diabetes.

### 2.6 Blood Collection and Assessment of the Effect of Aqueous Extract and Green Synthesized AgNPs on Biochemical Parameters

After the treatment period of 20 days with aqueous extract of *A. cepa* peels and AgNPs, all rabbits were euthanized by cervical dislocation and blood samples were collected from jugular veins in labeled blood collection tubes. The collected blood samples were allowed to clot for 30 min and then centrifuged (9300 × *g*, 4°C, and 30 min) to get clear serum for the assessment of blood sugar level, renal function tests, lipid profile and oxidative stress markers using commercially available Randox kits.

### 2.7 Statistical Analysis

The obtained data was evaluated by applying One‐way Analysis of Variance (ANOVA) using Statistical Package for Social Sciences (SPSS) (R) Version 26 for Windows. Difference was considered statistically significant at *p* < 0.05. The final results were presented as Mean ± standard deviation (Arise et al. 2023).

## 3 Results

### 3.1 Characterization

#### 3.1.1 LCMS‐MS Analysis

Full scanned mass spectral analysis using positive and negative electrospray ionization mode revealed the presence of 20 bioactive components. The identification of compounds was carried out on the basis of specific retention time, molecular mass and specific m/z values, which were then identified and compared with NIST reference library. Table 1 exhibits the major MS spectrum identified compounds and were phenolics, flavonoids, and glycosides along with their pharmacokinetic importance. Major peaks were observed by antidiabetic compounds with retention time ranges from 0.93 to 2.47 min. which were Quercitin, Chlorogenic acid, Myricitin, Vanillic acid, and Carnosic acid. Other identified compounds were Germacrene (0.91 min), Δ‐Cadinene (1.07 min), Cinnamaldehyde (0.47 min) and has anti‐microbial, Apigenin (1.28 min), Spirostanol (1.73 min), Luteolin (1.95 min), Nobiletin (2.55 min), Q‐3‐O‐Rhamnoside (3.28 min), Caffeic acid (1.00 min), Ferulic acid (1.11 min), Syringic acid (1.24), 7,8‐Dihydroxyflavone Hydrate (1.49 min), Retusin 8‐methyl ether (1.76 min), Kaempferol (1.86 min) and these compounds have antimicrobial, insecticidal, Anti‐inflammatory, anti‐bacterial potential, anti‐inflammatory, antioxidant, anti‐amyloidogenic, neuroprotective, cardioprotective, hepatoprotective, and renoprotective potential.

**TABLE 1:** Quantified phytoconstituents using LC–MS/MS chromatograms of *A. cepa* aqueous extract and their pharmacokinetic importance.

Sr. No.	m/z Ratio	MS/MS relative intensity	Retention time	Molecular mass	Compound name	Structure	Pharmacokinetic importance
1.	206	140.92, 144.92,163	0.91	205.20	Germacrene	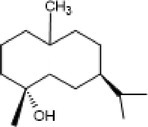	Anti‐microbial and insecticidal properties
2.	220.2	176.83, 187.0, 200	1.07	219.2	Δ‐Cadinene	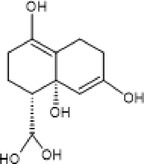	Anti‐inflammatory, anti‐bacterial
3.	117	70, 88, 98	0.47	116	Cinnamaldehyde	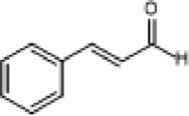	It possesses anti‐microbial
4.	271	88, 102, 106	1.28	270	Apigenin	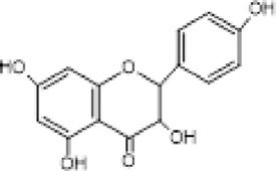	Helps in muscle relaxation and sedation, anti‐inflammatory, antioxidant, anti‐amyloidogenic, neuroprotective
5.	302.3	211, 228, 248	1.63	301.3	Quercetin	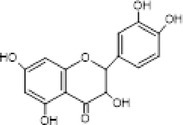	Antioxidant, anti‐inflammatory, anti‐diabetic, anti‐cancer
6.	254.3	266.8, 274.9, 277	1.73	253.3	Spirostanol	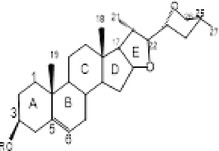	Cytotoxic effect
7.	366.3	245, 275, 305.17	1.85	354	Chlorogenic acid	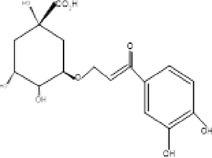	Anti‐obesity, inhibit development of liver steatosis, improve insulin sensitivity
8.	284.3	339, 351.1, 365.1	1.95	283	Luteolin	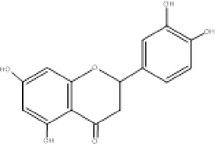	Anti‐inflammatory, anti‐allergy, anti‐cancer, pro‐oxidant
9.	319	254, 276, 289	2.31	318	Myricetin	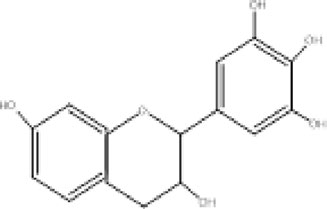	Anti‐oxidant, anti‐cancer, anti‐diabetic
10.	404.3	331.17, 343.17, 371.08	2.55	403	Nobiletin	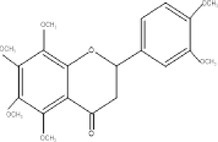	Therapeutic effects against neurological, inflammatory, cardiac, metabolic disorders, anti‐tumor
11.	448.5	285, 301, 321.17	3.28	447	Q‐3‐O‐Rhamnoside	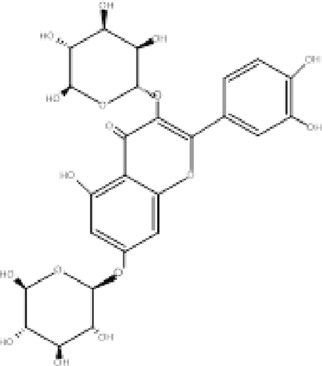	Cytotoxic effects
12.	465	366.8, 393, 421.1	3.51	464	Iso‐quercetin	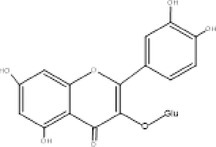	Anti‐diabetic, anti‐cancer, anti‐oxidants
13.	166	107.9, 123, 151	0.93	167	Vanillic acid	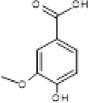	Anticancer, antiobesity, antidiabetic, antibacterial, anti‐inflammatory, and antioxidant
14.	178.2	135, 151, 161	1.00	179.2	Caffeic acid	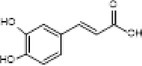	Anti‐carcinogenic, anti‐inflammatory, antioxidant
15.	194.2	135.9, 158.9, 177	1.11	195	Ferulic acid	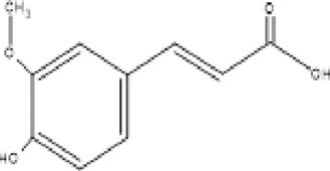	Prevent skin discoloration
16.	198.2	156.9, 181, 199	1.24	199	Syringic acid	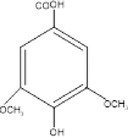	Help in preventing diabetes, CVDs, cancer, cerebral ischemia, anti‐endotoxic, hepatoprotective
17.	254.2	196.9, 237, 255.1	1.49	255	7,8‐Dihydroxyflavone Hydrate	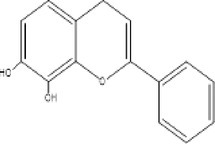	Neuroprotective effect
18.	268.2	255, 251, 269	1.76	269	Retusin 8‐methyl ether	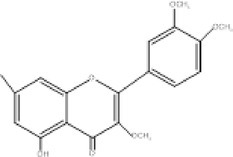	Antibacterial, antioxidant, anti‐inflammatory and antifungal activities
19.	286.2	228, 259, 285	1.86	287	Kaempferol	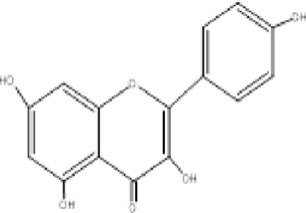	Antioxidant, anti‐inflammatory, antimicrobial, cardiovascular, and neuroprotective involve in hormones regulations
20.	330.2	259, 290, 299	2.47	331.2	Carnosic acid	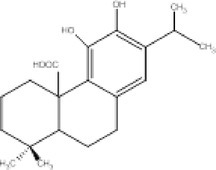	Prevent lipids from oxidation, anti‐obesity, neuroprotective

#### 3.1.2 Structural Analysis of AgNPs by X‐Ray Diffraction

**FIGURE 2:**
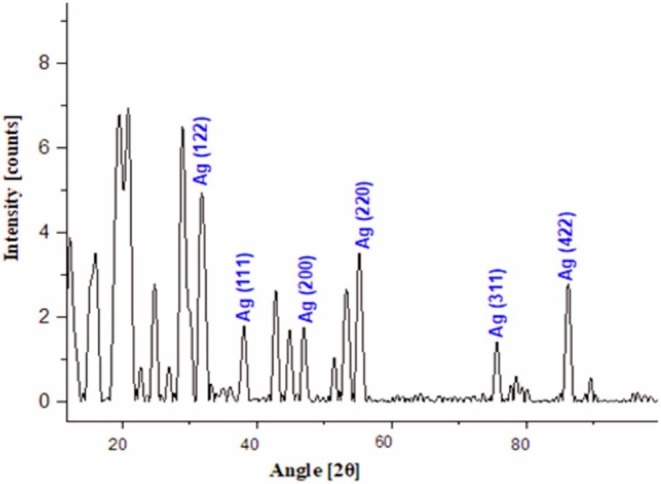
XRD spectral pattern of reduced AgNPs using aqueous extract of *A. cepa* peels.

X‐Ray diffraction analysis is considered as characteristic technique to accomplish the structural information of nanomaterials like phase nature, lattice parameters, and crystalline structure. The XRD spectral pattern of green synthesized AgNPs using aqueous extract of *A. cepa* peels is shown in Figure 2. The representation of very fine diffraction peaks was attributed to the crystalline nature of reduced AgNPs. Six distinct peaks were indexed at (122), (111), (200), (220), (311) and (422) with corresponding 2*ϴ* angles of 37.44°, 39.48°, 47.86°, 58.08°, 77.25°, 85.45°, respectively. The obtained data was well matched with the standard data of JCPDS file No. 03092 and reported. Table 2 gives the average crystalline size calculation and lattice planes. Using Debye‐Scherer equation, the average crystalline size of Ag‐Nanoparticles was calculated to be 20.09 nm. Sharp, intense peaks were the representation of the polycrystalline nature of AgNPs. Secondary peaks have also been observed, which are due to the presence of phytochemicals in *A. cepa peels* and act as capping agents for silver nanoparticles (AgNPs). Specific orientations of crystallites at 2*ϴ* angle confirmed the silver's face centered cubic structure. Lattice plane values indicate the biosynthesis of green synthesized AgNPs.

**TABLE 2:** Average crystalline size calculation and lattice planes.

Sr. No.	Peak position (2*θ*)	Theta (*θ*)	FWHM (*β*)	Crystallite size *D* (nm)	d‐Spacing (Å)	Lattice planes (hkl)
1.	37.44	18.72	0.99	8.47	2.40	1 2 2
2.	39.48	19.74	0.76	11.10	2.28	1 1 1
3.	47.86	23.93	0.46	18.89	1.89	2 0 0
4.	58.08	29.04	0.75	12.11	1.58	2 2 0
5.	77.25	38.625	0.34	29.90	1.23	3 1 1
6.	85.45	42.725	0.27	40.05	1.13	4 2 2

*Note:* Average crystalline size *D* (nm) of synthesized reduced Ag‐Nanoparticles = 20.09 nm.

### 3.2 Effect of Aqueous Extract and AgNPs on Biochemical Profile

Effects of different treatment doses on blood glucose level, lipid profile, and renal function tests in control and experimental groups are given in Table 3. After a single dose of Alloxan monohydrate injection, the blood glucose level, triglycerides, urea, creatinine, and cholesterol level were increased significantly (*p* < 0.05) in all the rabbits of experimental groups as compared to the negative control group treated with a normal diet. When these diabetes‐confirmed groups were subjected to treatment with different doses of green synthesized AgNPs, blood glucose level, triglycerides, urea, creatinine, and cholesterol level were significantly (*p* < 0.05) decreased (100 ± 0.8, 57.66 ± 0.8, 38 ± 0.8, 1.11 ± 0.08, 40 ± 0.00 mg/dL, respectively) at high dose (300 mg/mL per kg BW) as compared to the groups treated with the maximum dose (300 mg/mL per kg BW) of aqueous extract of *A. cepa* peels (154 ± 2.5, 67.5 ± 1.3, 51 ± 1.5, 2.6 ± 0.02, 53 ± 1.45 mg/dL, respectively). Alloxan monohydrate (5 mg/20 mL per kg BW) treated animals also showed significantly (*p* < 0.05) reduced levels of blood glucose, triglycerides, urea, creatinine, and cholesterol when compared with the positive control groups. Although the levels of urea, blood sugar, and cholesterol were significantly (*p* < 0.05) revived to near normal (36 ± 0.8, 36 ± 0.8, 42 ± 0.00 g/dL, respectively) in animals treated with the medium dose of reduced silver nanoparticles (200 mg/mL per kg BW) as compared to the positive control groups. The treatment of diabetic rabbits with the low dose (100 mg/mL per kg BW) of aqueous extract and AgNPs was not effective as observed by Group V and VIII. The influence of aqueous extract and AgNPs was comparable with Giblencelamide. The aqueous extract and AgNPs cause a sharp decrease in blood sugar levels which suggests their insulin stimulatory effect which is attributed to the partial regeneration of pancreatic cells (Ibikunle et al. 2022). Results from Perumalsamy et al. suggested that green synthesized silver nanoparticles using hydroethanolic extract of *Myristica fragrans* seeds show antidiabetic potential by inhibition of α‐amylase and α‐glucosidase activity and retarded glucose transport across the membranes (Perumalsamy and Krishnadhas 2022).

**TABLE 3:** Effect of aqueous extract and AgNPs on blood sugar level, lipid profile and renal function.

Groups	Blood glucose (mg/dL)	Lipid profile		Renal function test	
		Triglycerides (mg/dL)	Cholesterol (mg/dL)	Urea (mg/dL)	Creatinine (mg/dL)
Group I Negative control group	98 ± 1.6^ab^	56.2 ± 2.0^ab^	39.3 ± 1.7^ab^	38 ± 1.1^ab^	1.9 ± 0.01^ab^
Group II Positive control group	275 ± 2.4^aa^	143 ± 1.4^aa^	66.2 ± 2.0^aa^	75 ± 1.54^aa^	2.9 ± 0.01^aa^
Group III Drug control group	97 ± 0.8^ab^	56.66 ± 0.8^ab^	39 ± 0.00^ab^	37.66 ± 0.8^ab^	1.10 ± 0.00^ab^
Group IV Aqueous extract (100 mg/mL per kg BW)	198 ± 1.8^aa^	102.2 ± 1.5^aa^	62 ± 2.1^aa^	57 ± 2.1^aa^	2.7 ± 0.03^aa^
Group V Aqueous extract (200 mg/mL per kg BW)	176 ± 2.1^aa^	75 ± 1.8^aa^	58 ± 1.9^aa^	55 ± 1.7^aa^	2.66 ± 0.01^aa^
Group VI Aqueous extract (300 mg/mL per kg BW)	154 ± 2.5^aa^	67.5 ± 1.3^aa^	53 ± 1.45^aa^	51 ± 1.5^aa^	2.6 ± 0.02^aa^
Group VII AgNPs (100 mg/mL per kg BW)	184 ± 3.2^aa^	97.5 ± 1.9^aa^	59 ± 1.3^aa^	53 ± 1.9^aa^	2.59 ± 0.01^aa^
Group VIII AgNPs (200 mg/mL per kg BW)	105 ± 0.8^ab^	59.66 ± 1.5^ab^	42 ± 0.00^ab^	36 ± 0.8^ab^	1.13 ± 0.08^ab^
Group IX AgNPs (300 mg/mL per kg BW)	100 ± 0.8^ab^	57.66 ± 0.8^ab^	40 ± 0.00^ab^	38 ± 0.8^ab^	1.11 ± 0.08^ab^

*Note:* Values are expressed as Mean ± SE (standard error) of means of study groups. Letters in superscript indicates statistical ^aa^significant and ^ab^non‐significant mean differences at *p* < 0.05 among various groups (*n* = 5).

### 3.3 Effects of Aqueous Extract and AgNPs on Major Antioxidant Enzymes

Oxidative stress is a major cause of diabetes. Studies have reported that Alloxan monohydrate is an active mediator of reactive oxygen species which disturbs the major antioxidant enzymes as well as causes β‐cells toxicity resulting in diabetes (im Walde et al. 2002). In present investigation, Alloxan monohydrate (160 mg/ml per kg BW) induces diabetes which is clearly evidenced by the significant (*p* < 0.05) decrease in the activities of enzymatic antioxidants like CAT, SOD, and GPx in Group II rabbits (1.5 ± 0.01, 3.2 ± 0.01, 698 ± 1.8 IU/mL, respectively) as compared to Group I rabbits (3.9 ± 0.02, 6.2 ± 0.01, 920 ± 1.7 IU/mL, respectively). Effect of different treatment doses on serum oxidative stress biomarkers in control and experimental groups are given in Table 4. Compared to positive control (diabetic) Group II rabbits, the levels of Glutathione peroxidase were significantly (*p* < 0.05) reduced to normal at high dose concentration (300 mg/mL per kg BW) of both aqueous extract of *A. cepa* peels (820 ± 2.2 IU/mL for Group VI) and AgNPs (918 ± 2.0 IU/mL for Group IX). It was observed that treatment of diabetes confirmed rabbits with low (100 mg/mL per kg BW) and medium dose (200 mg/mL per kg BW) of green synthesized AgNPs showed significant (*p* < 0.05) recovery of serum antioxidant enzymatic status (2.6 ± 0.01, 5.4 ± 0.02, 823 ± 1.7 IU/mL for Group VII and 3.4 ± 0.08, 5.9 ± 0.08, 912 ± 2.4 IU/mL for Group VIII respectively) as compared to the rabbits of Group II. Although no significant difference was observed in the levels of enzymatic antioxidants, that is, GPx, SOD, and CAT between the rabbits of drug control Group III and negative control Group I.

**TABLE 4:** Effects of aqueous extract and AgNPs on major anti‐oxidant enzymes.

Groups	Catalase (CAT) (IU/mL)	Superoxide dismutase (SOD) (IU/mL)	Glutathione peroxidase (GPx) (IU/mL)
Group I Negative control group	3.9 ± 0.02^ab^	6.2 ± 0.01^ab^	920 ± 1.7^ab^
Group II Positive control group	1.5 ± 0.01^aa^	3.2 ± 0.01^aa^	698 ± 1.8^aa^
Group III Drug control group	3.8 ± 0.08^ab^	6.1 ± 0.08^ab^	920 ± 2.3^ab^
Group IV Aqueous extract (100 mg/mL per kg BW)	2.2 ± 0.03^aa^	4.7 ± 0.01^aa^	754 ± 2.1^aa^
Group V Aqueous extract (200 mg/ml per kg BW)	2.7 ± 0.01^aa^	4.9 ± 0.02^aa^	788 ± 1.9^aa^
Group VI Aqueous extract (300 mg/mL per kg BW)	2.9 ± 0.02^aa^	5.1 ± 0.01^aa^	820 ± 2.2^aa^
Group VII AgNPs (100 mg/mL per kg BW)	2.6 ± 0.01^aa^	5.4 ± 0.02^aa^	823 ± 1.76^aa^
Group VIII AgNPs (200 mg/mL per kg BW)	3.4 ± 0.08^ab^	5.9 ± 0.08^ab^	912 ± 2.4^ab^
Group IX AgNPs (300 mg/mL per kg BW)	3.6 ± 0.08^ab^	6.0 ± 0.08^ab^	918 ± 2.0^ab^

*Note:* Values are expressed as Mean ± SE (standard error) of means of study groups. Different letters in superscript indicates statistical ^aa^significant and ^ab^non‐significant mean differences at *p* < 0.05 among various groups (*n* = 5).

## 4 Discussion

Diabetes mellitus is a major health problem and classified into type I and type II diabetes on the basis of induction mechanism. Type I is characterized by a decrease in insulin production due to a reduction in pancreatic functional activity. Although type II is mediated by the prevalence of glucose intolerance and the peripheral cells' decreased sensitivity to insulin. Both types lead to hyperglycemia due to the compromised uptake of glucose by the insulin targets in the periphery (Federiuk et al. 2004; Kashif et al. 2023; Mughal et al. 2024). In the current study, diabetes was induced by a single dose of Alloxan monohydrate, and its effects were studied. Further, it aimed at finding the inhibitory potential of aqueous extract of *A. cepa* peels and silver nanoparticles against Alloxan induced diabetes in rabbits.

The physical characterization of green synthesized AgNPs satisfies the antidiabetic potential of nanodrug. XRD analysis confirmed the crystalline nature of AgNPs which classifies it as an efficient nanodrug. Mass spectral analysis of aqueous extract of *A. cepa* peels yielded a fine spectrum of 20 bioactive components in the form of phenolics, flavonoids, and glycosides, responsible for the reduction and stabilization of AgNPs. A published study of Abdelmoaty et al. (2010) reported the antidiabetic potential of Quercitin at the dose concentration of 15 mg/mL per kg BW by significantly normalizing the antioxidant enzyme activities (GPx, SOD, and CAT) on streptozotocin induced diabetes in rats. In vitro studies by Singh et al. (2021) have evidenced that Chlorogenic acid at the dose concentration of 30 μg/mL per kg BW significantly suppressed the elevated levels of blood glucose, triacylglycerides, glutamic oxaloacetic transaminase, glutamic pyruvic transaminase, γ‐glutamyl transferase, alkaline phosphatase, total bilirubin, creatinine, urea, and uric acid in diabetic rats. In view of the antioxidant potential of vanillic acid, the antidiabetic effect of vanillic acid coated silver nanoparticles was evaluated by Alamri et al. who concluded that the synthesized AgNPs showed stronger defense against streptozotocin induced diabetes in rats as compared to only treatment with vanillic acid (Alamri and El Rabey 2024).

From our current study results, it was observed that Alloxan monohydrate (5 mg/20 mL per kg BW) treated animals showed significantly (*p* < 0.05) reduced levels of blood glucose, triglycerides, urea, creatinine, cholesterol and down‐regulated the levels of antioxidant enzymes when compared with positive control groups. Alloxan monohydrate undergoes a redox reaction with beta cells and causes overproduction of reactive oxygen species like superoxide radicals and hydrogen radicals. Alloxan monohydrate causes the reduction of glutathione, which is an antioxidant. Overproduction of reactive oxygen species and reduction in antioxidants causes higher oxidative stress and consequently necrosis or apoptosis of the beta cells of the Islet of Langerhans (Lenzen 2008). Alloxan monohydrate attacks beta cells and destroys them. Due to the low level of insulin production, glucose levels increase in the bloodstream, resulting in hyperglycemia (Trivedi et al. 2004), which is also shown in our study in alloxanized rabbits. Increased levels of glucose damage the blood vessels and glomeruli, which in turn causes high levels of creatinine, urea and cholesterol in diabetic rabbits as compared to positive control (non‐diabetic) rabbits. Published study of Aja et al. (2015) reported an increased level of urea in experimental animals after induction of diabetes through Alloxan monohydrate; hence, it supports the results of our current study.

The results from treatment of animals with varied doses of aqueous extract of *A. cepa* peels and AgNPs found an overall recovery of diabetes induced by Alloxan monohydrate. Maximum recovery was shown by high dose of silver nanoparticles, whereas minimum improvement was observed in low dose of aqueous extract administered animals. Silver nanoparticles due to their smaller sizes enter into the cell and enhance their capability to produce more insulin to eliminate diabetes. Similar results were also observed, when Alloxan induced diabetic animals were treated only with aqueous extract of *Allium cepa* peels (Lee et al. 2014). In diabetic condition, level of anti‐oxidant enzymes, such as catalase, superoxide dismutase, and glutathione peroxidase were decreased due to the over production of ROS. It leads to alleviation in lipid peroxidation and oxidative stress (Ozcan et al. 2004). When rabbits were treated with aqueous extract of *A. cepa* peels and silver nanoparticles, there were significant increases in the level of anti‐oxidant enzymes due to the presence of phytochemicals having anti‐oxidant potential. Flavonoids of *A. cepa* peels donate electrons to free radicals and neutralize them, which prevent oxidative damage to cells. Few phytochemicals present in onion peels, stimulate the production of anti‐oxidant enzymes, or regulate the expression of enzymes like catalase, superoxide dismutase, and glutathione peroxidase. Allyl sulfide, a sulfur containing compound present in onion peels, stimulate the production of glutathione, which is a crucial anti‐oxidant, regulating the functions of different anti‐oxidant enzymes. Different phenolics and flavonoids present in *Allium cepa* peels, directly reduces the oxidative stress, which indirectly supports the activity of ani‐oxidant enzymes (Lee et al. 2014).

## 5 Conclusion

In conclusion, this research works advances our knowledge about the synthesis of silver nanoparticles using *A. cepa* peels as a stabilizing and reducing function. Characterization through XRD analysis revealed 20.9 nm size nanoparticles. LCMS‐MS analysis revealed the presence of about 20 bioactive compounds having therapeutic potential including antidiabetic activities. Extraction of bioactive phytoconstituents from *A. cepa* peels aqueous extract and their pharmacokinetic importance proclaims that specific protocols should be introduced and established for extraction and isolation of these therapeutic compounds like quercetin, anthocyanins, kaempferol, ferulic acid, and chlorogenic acid from *A. cepa* peels and synthesis of AgNPs in the formulation of antidiabetic drugs. The study demonstrated remarkable antidiabetic potential of *A. cepa* peels extract and the synthesized silver nanoparticles as evidence through the decreased glucose, cholesterol and triglyceride levels while increased antioxidant enzymes activities in *A. cepa* extract and synthesized nanoparticle treated animals. However, more in‐depth in vitro and in vivo studies are required to establish synthesized AgNPs using *A. cepa* peels as a promising competitor for the treatment of type 2 diabetes mellitus with high therapeutic efficiency and lower side effects.

## Author Contributions

**Sadia Batool:** data curation (equal), formal analysis (equal), investigation (lead), methodology (lead), visualization (equal), writing – original draft (equal). **Mazhar Abbas:** conceptualization (lead), funding acquisition (equal), project administration (lead), resources (equal), supervision (lead), validation (equal). **Sidra Ashraf:** conceptualization (supporting), formal analysis (equal), validation (equal), writing – original draft (supporting). **Muhammad Kamran Rafique:** data curation (supporting), formal analysis (equal), investigation (equal), software (supporting), visualization (supporting). **Aqsa Mumtaz:** data curation (supporting), methodology (equal), visualization (supporting), writing – original draft (equal). **Tariq Hussain:** conceptualization (supporting), investigation (equal), resources (equal), validation (equal), writing – review and editing (equal). **Muhammad Riaz:** formal analysis (equal), software (supporting), visualization (equal), writing – review and editing (lead). **Ghulam Rasool:** formal analysis (equal), validation (equal), visualization (supporting), writing – review and editing (supporting). **Muhammad Adnan Ayub:** investigation (supporting), software (supporting), validation (equal), writing – review and editing (supporting). **Quzi Sharmin Akter:** formal analysis (equal), validation (supporting), visualization (supporting), writing – review and editing (equal).

## Ethics Statement

The study plan was approved by the research scrutiny committee/Institutional Review Board (IRB), College of veterinary and Animal Sciences, Jhang campus, University of Veterinary and Animal Sciences (UVAS), Lahore, Pakistan.

## Conflicts of Interest

The authors declare no conflicts of interest.

## Data Availability

The data will be available from Principal and Corresponding authors on reasonable request.
